# Optimal micro-grid battery scheduling within a comprehensive smart pricing scheme

**DOI:** 10.1038/s41598-025-02690-9

**Published:** 2025-06-20

**Authors:** Mohammed Ashraf Ali, Ahmad H. Besheer, Hassan M. Emara, Ahmed Bahgat

**Affiliations:** 1 Electrical Power Engineering Department, Faculty of Engineering, Cairo University, Giza, Egypt; 2 Environmental Studies and Research Institute, University of Sadat City, Sadat City, Egypt

**Keywords:** Smart grid, Optimal schedule, LiFePO4 degradation, Demand charge, Dynamic programming, Batteries, Energy grids and networks, Power distribution, Photovoltaics

## Abstract

The challenge of optimizing battery operating revenue while mitigating aging costs remains inadequately addressed in current literature. This paper introduces a novel cost–benefit approach for scheduling battery energy storage systems (BESS) within microgrids (MGs) that features smart grid attributes. The proposed comprehensive approach accounts for fluctuations of real-time pricing, demand charge tariffs, and battery degradation cost. Using the dynamic programming technique, a novel high-speed BESS scheduling optimization algorithm that incorporates a LiFePO4 battery degradation cost model is developed, achieving substantial monthly operational cost savings for the MG with a fine-grained sampling interval of nine minutes and execution time under one minute. The algorithm utilizes day-ahead forecasts for MG load profiles and photovoltaic output power, enabling the prediction of BESS’s optimal power profile a day in advance. The algorithm’s rapid execution enables real-time adaptability, allowing BESS scheduling to dynamically respond to grid fluctuations. The proposed approach outperforms existing methods in the literature, delivering MG operational cost savings ranging from 33.6% to 94.8% across various scenarios. Consequently, this approach enhances MG operational efficiency and provides significant cost savings.

## Introduction

Continuous stress of increasing energy demand and prices necessitates further electrical power generation and consumption improvement. Increasing the penetration of renewable energy and using grid-connected storage systems on the generation side can be considered a technically viable solution. On the other hand, consuming energy more efficiently and implementing demand response programs are generally applicable methods on the consumption side. Integrating all these methods in a shared environment can benefit consumers and utilities substantially. Such environments include smart homes that remotely monitor or control home appliances and local energy-generating units [1]. In this environment, incorporating renewable energy resources such as wind turbines and solar panels supported by storage systems makes the power system efficient, reduces peak-to-average load, minimizes the cost of production, increases the reliability of the system, and balances the grid [2].

The smart grid has been developed using recent communication technologies (Zigbee, WiMAX, PLC, etc. [3]) that enable bidirectional data and power flow. These capabilities now support electric vehicle integration through: distributed consensus algorithms for grid-station power sharing [4], solar-hybrid charging station scheduling [5], and techno-economic-environmental nexus optimization [6]—all leveraging the smart grid’s real-time control infrastructure. In response to such evolution, smart pricing schemes have been created to fulfill the requirements of modern power systems. Some of these schemes are real-time pricing (RTP), time of use (ToU), and critical peak pricing (CPP), which are time-based pricing schemes. These smart pricing schemes play a crucial role in demand response so that the system operates efficiently [7]. Moreover, the demand charge tariff (DCT), where the utility charges consumers a monthly penalty proportional to their recorded peak demand this month, is also introduced to complement these schemes [8, 9].

Therefore, to achieve maximum energy utilization in microgrids (MGs) while keeping serving the loads as a priority, battery energy storage systems (BESS) should absorb energy from photovoltaic (PV) units during periods of excess solar output power or from the grid at low tariff period and release energy during load peaks or high tariff periods. Hence, it is essential to rely on short-term load forecasting (STLF) to predict load and PV output power on an hourly or less basis for up to one week. Considering RTP variation, this allows scheduling the battery’s optimal power profile (absorbing/releasing) to achieve the minimum microgrid (MG) operation cost per day [10]. Many researchers proposed various approaches for STLF aiming to minimize forecasting errors that serve BESS optimal scheduling [2, 11–14].

In this regard, Lithium-ion (Li-ion) batteries are superior candidates for grid storage applications because of their high power density and higher cycle life than other battery types. Besides, their manufacturing cost is expected to continue decreasing [15].

B. Lian et al. [16] explored the potential of using LiFePO4 battery in grid storage applications due to its prime cycle life and reliability. LiFePO4 can operate under a wide state of charge (SOC) range and extended life cycle for deep and shallow cycling. To study the operating cost of these Li-ion batteries, M. Badawy et al. [17] stated three main factors that impact batteries’ degradation cost. These three factors are the battery temperature, the average state of charge, and the depth of discharge (DOD). Then, the overall battery degradation cost equals the maximum cost from these three factors. Similarly, the capacity fading effect will also be calculated based on the worst impact of these three factors.

Various approaches have been studied to propose the optimal schedule for BESS. Recently, Zia et al. [18] proposed a PV-BESS integrated with an internet of things (IoT)-based monitoring prototype [19] to enhance energy management for residential and EV loads under a ToU pricing scheme. Their system, evaluated using HOMER Grid software, explored two scenarios: domestic loads only and combined domestic and EV loads. The results showed substantial reductions in both electricity costs and carbon emissions, particularly in the presence of EVs. The study effectively demonstrated the role of IoT in enabling real-time system visibility and improved decision-making. In a parallel recent development, Hossain et al. [20] demonstrated a hybrid optimization approach for PV-BESS peak shaving in commercial buildings, combining rule-based control with genetic algorithm (GA) optimization. Their method achieves 49.8% peak demand reduction through dynamic demand limits while maintaining 50% SOC for operational flexibility. Building on sizing optimization, Hossain et al. [21] addressed optimal sizing of PV-BESS systems for Malaysian commercial buildings, proposing a rule-based energy management strategy coupled with particle swarm optimization (PSO). Their work demonstrates how 32 kW PV/14kWh BESS configurations can reduce electricity costs by 12.33% while addressing rooftop space constraints and feed-in tariff (FIT) limitations under fixed demand and energy tariffs. B. Jeddi et al. [22] developed a home energy management system (HEMS) integrating grid, BESS, and PV with day-ahead load profiles having one-hour sampling. Their cost function incorporated varying billing rates (constant/ToU/dynamic), FIT, and battery degradation (modeled via charge cycles and DOD). Using dynamic programming (DP), they discretized the problem into recursive sub-problems for optimal SOC solutions. With Q. Wei et al. [23], an iterative control law sequence of the battery power is obtained through adaptive dynamic programming (ADP). They carried out the numerical comparison among other techniques such as time-based Q-learning (TBQL), PSO and model predictive control (MPC). This comparison verifies the superiority in cost minimization of the developed ADP-based algorithm over the other compared techniques. Y. Zhang et al. [24] employed infinity norm minimization to dispatch limited-capacity energy storage for reducing net load variance, considering flattening levels, storage capacity, and deployment across voltage-constrained distribution feeders. K. Abdulla et al. [25] considered a dynamic multi-factor degradation model and contributed to extending batteries’ lifetime value by 160% relative to other basic setpoint-controlled operations of many different systems. K. Morrissey et al. [26] scheduled BESS for load leveling and ramp-rate control using day-ahead load and PV forecasts, ensuring voltage and SOC constraints, with end-of-day SOC equal to or greater than the beginning, on a 6.5 MW system with energy resources at different nodes. In [27], B. Jeddi et al. reduced optimization time (30 secs for hourly samples) using a differential model with adjustable corridor width: wider corridors approach exact DP solutions at higher computational cost, while narrower ones sacrifice accuracy for speed. The method outperforms mixed-integer nonlinear programming (MINLP), Q-learning, and ADP in efficiency and enables intra-day adjustments for load/PV forecast uncertainties. Y. Li et al. [28] developed a DP-based operation strategy for grid-connected PV-BESS, optimizing real-time energy flow under ToU pricing to maximize revenue. The strategy minimizes net present value (NPV) over a typical year while considering battery aging, demand response, and PV self-consumption. Sensitivity analysis identified electricity price as the most critical parameter for system economics. Finally, yet importantly, F. Luo et al. [29] worked on the day-ahead forecasts of PV and non-deferrable load profiles with ten-minute sampling. A natural aggregation algorithm (NAA) is proposed to optimally schedule dependent loads and the BESS with a cost function regarding RTP and DCT. A comparison among other bio-inspired optimization algorithms is performed, resulting in a significant lead in performance for the NAA. Those other algorithms are PSO and differential evolution (DE). Their optimization program was implemented in Matlab using a workstation with 128 gigabytes (GB) of memory and two Intel Xeon processors. However, the execution time exceeded an hour for the minimum proposed solution population size.

With all the relevant research mentioned above, Table 1 summarizes their work, highlighting all their achievements and limitations and reflecting the following remark.

**Table 1:** Literature Summary of BESS Scheduling.

References	Considered Cost Terms	Achievements	Limitations	Technique
[18]	ToU	Reductions in energy cost and carbon emissions	Disregarding RTP, DCT & batteries degradation Large sampling period (one hour)	Based on HOMER Grid software
[22, 28]	ToU, RTP, FIT and battery degradation		Disregarding DCT Large sampling period (one hour)	DP
[25]		Extending batteries’ lifetime by 160%		Stochastic DP
[27]		Optimization execution time is 30 s		Differential DP
[23]	RTP and battery degradation	Surpassing TBQL, PSO and MPC techniques	Disregarding DCT	ADP
[24]	DCT		Disregarding RTP Disregarding batteries degradation	Infinity norm minimization
[26]				Generic algorithm
[20]				Hybrid (Rule-based + GA)
[29]	RTP and DCT	Low sampling period (10 min) Surpassing PSO and DE techniques	The BESS scheduling is not optimal Long execution time (one hour) Disregarding batteries degradation	NAA

### Remark

The problem of MG optimal battery scheduling has not been thoroughly studied, considering the simultaneous DCT, RTP, and battery degradation costs.

- Cost due to DCT is not considered in [18, 22, 23, 25, 27, 28].
- Cost due to RTP is not considered in [18, 20, 24, 26].
- The battery degradation cost is not considered in [18, 20, 24, 26, 29].

This paper proposes an MG BESS scheduling algorithm based on the DP optimization technique that simultaneously considers RTP, DCT and battery degradation replacement costs, based on predefined load and PV forecast data. The scheduling module decides the optimal battery power value and flow direction (absorbing/releasing) for the entire day ahead. The superiority of the developed algorithm is validated against the work done in [29], which optimizes RTP and DCT cost terms in a single cost function. Additionally, the developed algorithm also incorporates the battery degradation effect to achieve maximum utilization efficiency with minimal operational costs for the MG. Furthermore, a sensitivity analysis is performed to study the variation of different input parameters on the final cost. These parameters are BESS capacity, initial and final SOC, and algorithm calculation step.

The main contribution of this paper can be summarized as follows:

1. Optimization of a novel and comprehensive cost function is proposed that includes not only both the smart pricing terms of RTP and DCT but also the battery degradation replacement cost.
2. A reduced sampling period (nine minutes) is adopted to detect load peaks and apply shaving if necessary.
3. The developed algorithm is not time-consuming. It can perform the whole day-ahead scheduling optimization in one minute. This allows the online operation to accommodate any scheduling change with the updated input predicted profiles.
4. The achieved optimized cost is better than that reported in [29] under the same operating conditions.

The rest of the paper is organized as follows: Section “Methodology and approach” presents the methodology of the proposed approach. Section “Description of the case study” demonstrates the selected case study with all its parameters and conditions. Section “Results and discussion” demonstrates the application of the proposed algorithm in various scenarios, discusses the results, and performs a sensitivity analysis of the effect of different parameters. Finally, conclusions and future work are drawn in Section “Conclusions and future research”, and a proposal is presented for bridging the gap between theoretical optimization and practical demands.

## Methodology and approach

### A. Overview of the MG energy management environment

**Fig. 1:**
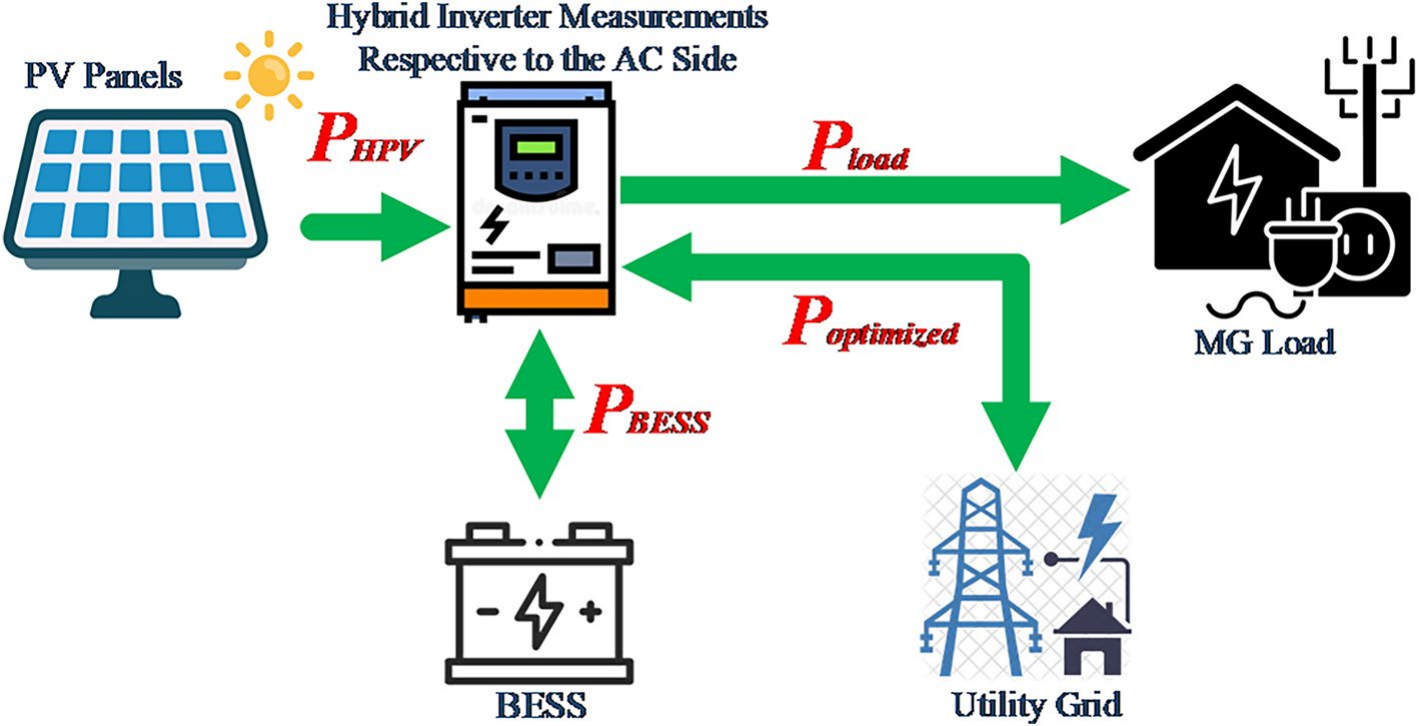
MG topology of hybrid PV inverter, utility grid, and BESS.

The studied MG topology in Fig. 1 consists of the utility grid, a PV hybrid inverter, solar PV modules, an energy storage system, and loads. Figure 1 shows how the hybrid inverter enables bidirectional power flow between the utility grid and battery storage. The battery storage can be charged from the output PV power or the utility grid consumed power.

This hybrid PV inverter can receive a reference signal from a supervisory level through data communication to set the value and direction of the battery’s operating power and energy flow. It can also send real-time battery SOC measurements, which are stored in its memory through communication.

**Fig. 2:**
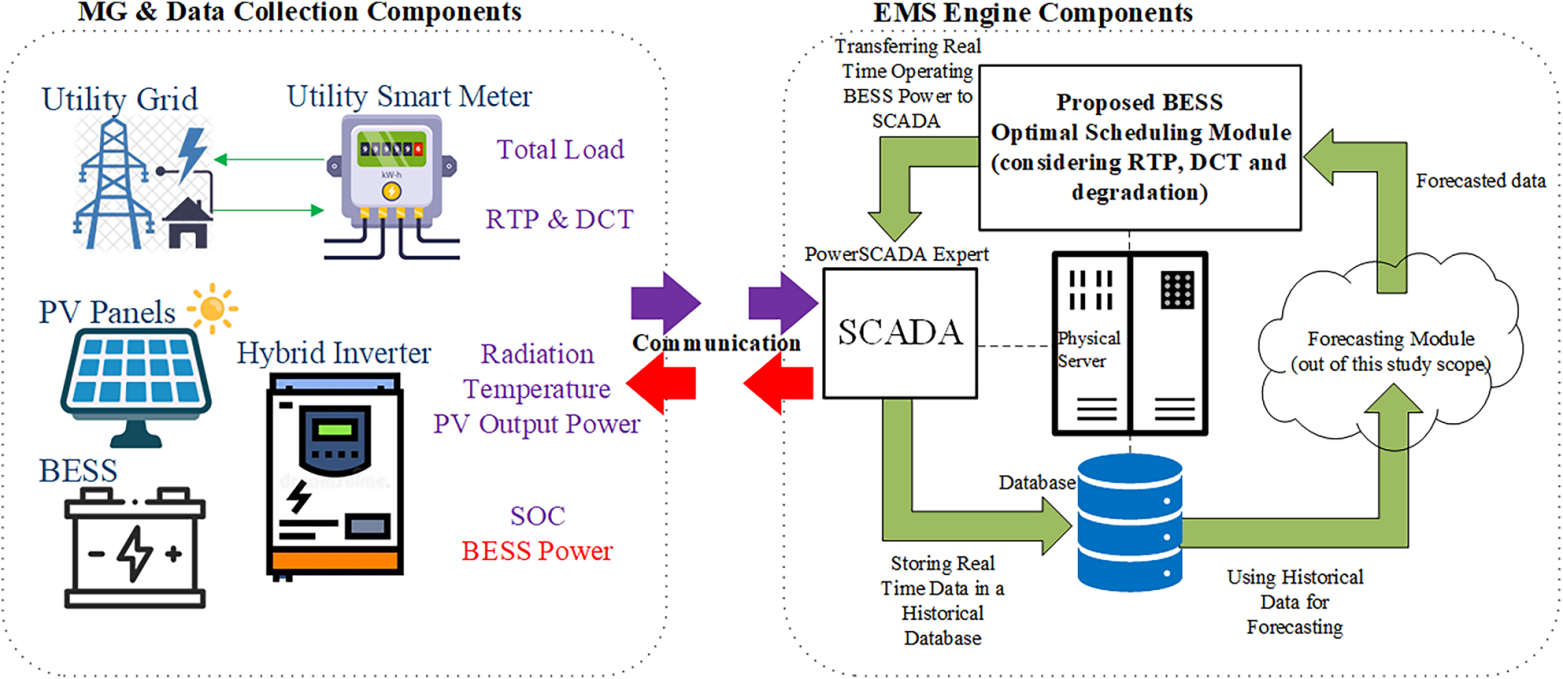
Schematic of the proposed MG energy management system.

The developed MG energy management scheme in this work, as shown in Fig. 2, is integrated with the previously developed supervisory control and data acquisition (SCADA) platform by the authors [31, 32]. Using the SCADA input/output (IO) server, this SCADA platform is responsible for wired/wireless communication to receive/send all MG measurements and setpoints among all meters and the PV inverter using the Modbus TCP protocol. It cyclically reads and writes measurements and setpoints every ten seconds. For example, in real time, SCADA reads total power and utility tariffs from the utility meter. It also reads the real-time PV output power, radiation, and temperature from the hybrid PV inverter.

Based on [29], the forecasting module ideally uses a long-term historical database to predict the day-ahead load profile with a reasonable sampling period to study peak shaving (9 min). It also determines the peak shaving threshold below which there will not be any additional load shaving. As in (1), this shaving threshold limit is the maximum of the previously recorded peak and the upcoming predicted peak in the running operation month after subtracting the BESS discharging rated power.

1 $$P_{shaving\_limit} = {\text{ Max }}\left( {P_{optimized\_his} ,{ }P_{feeder\_peak\_f} - \left| {P_{BESS\;min} } \right|} \right)$$

Based on all collected and stored PV data, it predicts the PV output power profile with the same sampling period to accommodate rapid sun shading effects. It also predicts the day-ahead RTP profile with the same sampling period and proposes the DCT constant if not given. To meet the following day’s requirement, it is responsible for adjusting ($$SO{C}_{end})$$. The BESS scheduling module should achieve this SOC at the end of the operating day.

All predicted profiles serve as inputs to the BESS scheduling module, which calculates the projected BESS power profile through the end of the day. Finally, the BESS scheduling module sends the BESS current operating power and flow direction to the SCADA engine to let its IO server send it to the PV inverter through communication.

### B. Problem formulation

Given a particular load curve for the MG shown in Fig. 1, which is connected to the utility feeder, determine the optimal BESS power profile (${P}_{BESS}(t)$). This profile reflects the batteries’ optimal charging/discharging pattern throughout the day, such that the electricity cost function in (2) is minimized, and all other constraints in (3)-(15) are respected. The smart pricing costs due to RTP and DCT are considered in (2), (6), (7) using the cost terms of (${C}_{Energy}$) and (${C}_{Demand}$). The formulated BESS degradation model in (2), (8) has the cost term of (${C}_{BESS}$), which is discussed in [17, 22, 33]. This degradation mathematical model formulates the total degradation cost (8) as the summation of all costs at each time slot. Each time slot cost is proportional to the magnitude of BESS operating power at this slot and inversely related to the BESS number of cycles and DOD.

BESS operating power and SOC should be constrained as in (3), (4). At the end of the working day, BESS SOC should be greater than or equal to an adjustable setpoint $(SO{C}_{end})$ as defined in (5) to meet the following day’s load requirements. Due to the lack of operational data, DOD is calculated as in (9). SOC is calculated in (10) based on the degraded capacity (${E}_{BESS D\_capacity}$). Degraded capacity in (12) is decremented in each time slot by a factor that is inversely related to the batteries’ life cycles ($N\left(t\right)$). Moreover, BESS’s expected life cycles versus DOD is represented in (13) as in [33]. Finally, a time series of the net power profile after applying the optimized BESS power (${P}_{optimized}(t)$) is defined in (14), which shows an optimal active power dispatch for different micro-grid assets along the day to serve the input predicted load curve for this working day. The MG feeder load curve is defined as in (15). MG feeders’ losses are included in $({P}_{load}\left(t\right)$).

2 $$Mininize\left( {Cost\left( {P_{optimized} \left( t \right)} \right)} \right) = Mininize\left( {C_{Energy} + C_{Demand} + C_{BESS} } \right)$$

Subjected to:

3 $${{P}_{BESS}}_{min}\le {P}_{BESS}(t)\le {{P}_{BESS}}_{max}$$

4 $$SO{C}_{min}\le SOC(t)\le SO{C}_{max}$$

5 $$SOC(24)\ge SO{C}_{end}$$

Such that:

6 $$C_{Energy} = \mathop \sum \limits_{t = 0}^{24} \left( {P_{optimized} \left( t \right){*}\Delta T{*}RTP\left( t \right)} \right)$$

7 $${C}_{Demand}=Max\left({P}_{optimized}\left(t\right), {P}_{optimized\_his} \right)*DCT$$

8 $$C_{BESS} = \mathop \sum \limits_{t = 0}^{24} \left( {\frac{{C_{Initial} {*}\left| {P_{BESS} \left( t \right)} \right|{*}\Delta T}}{{2{ }N\left( t \right){*}DOD\left( t \right){*}E_{{BESS{ }D\_capcity}} \left( t \right){*}\mu_{ch - disch}^{2} }}} \right)$$

9 $$DOD\left(t\right)=1-SOC(t)$$

10 $$SOC\left(t\right)={E}_{BESS}(t)/{E}_{BESS D\_capacity}(t)$$

11 $${E}_{BESS}\left(t\right)={E}_{BEES}\left(t-1\right)+{P}_{BESS}(t)*\Delta T*{\mu }_{ch-disch}$$

12 $${E}_{BESS {D}_{capacity}}\left(t\right)={E}_{BESS {D}_{capacity}}\left(t-1\right)-{E}_{BESS nominal}*\Delta t/N(t-1)$$

13 $$N\left(t\right)=a*{\left(DOD\left(t\right)\right)}^{b}*\text{exp}(c*DOD\left(t\right)) \forall a\ge 0, b\&c\le 0$$

14 $${P}_{optimized}(t)={P}_{feeder}(t)+{P}_{BESS}(t) \forall 0\le t\le 24$$

15 $${P}_{feeder}\left(t\right)={P}_{load}\left(t\right)-{P}_{HPV}\left(t\right)$$

In this paper, BESS is treated as a load, i.e., its optimized power profile has a positive sign value during charging and a negative sign value during discharging.

**Fig. 3:**
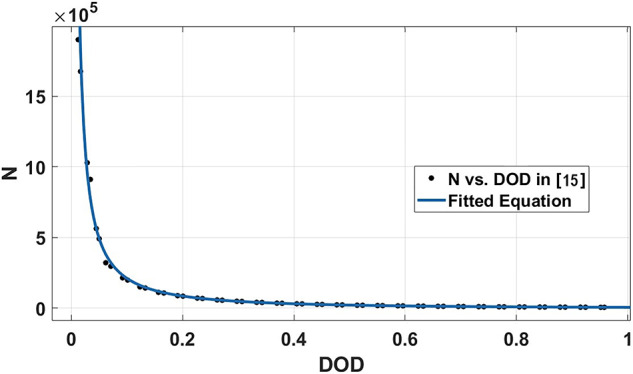
LiFePO4 life cycles versus DOD.

The energy storage technology in this work is chosen to be LiFePO4 due to its versatility. The constants for the LiFePO4 life cycles (N) versus DOD equation in (13) are derived from the curve-fitted data in [15], as shown in Fig. 3. LiFePO4 pack prices dropped by 14% from 2022 levels, reaching a low record of $139/kWh this year [34]. This reduction was driven by decreasing raw material and component prices, as well as increased production capacity. So, ($${C}_{Initial cost}$$) will equal ($${\text{\$}140/kWh * E}_{BEES nominal}$$).

### C. Solution environment and approach

As previously demonstrated, the schematic of the proposed MG energy management system in Fig. 2 shows the integration environment of the scheduling algorithm along with the SCADA system, database, and forecast data all in one physical machine based on multi-core and multi-thread processors.

**Fig. 4:**
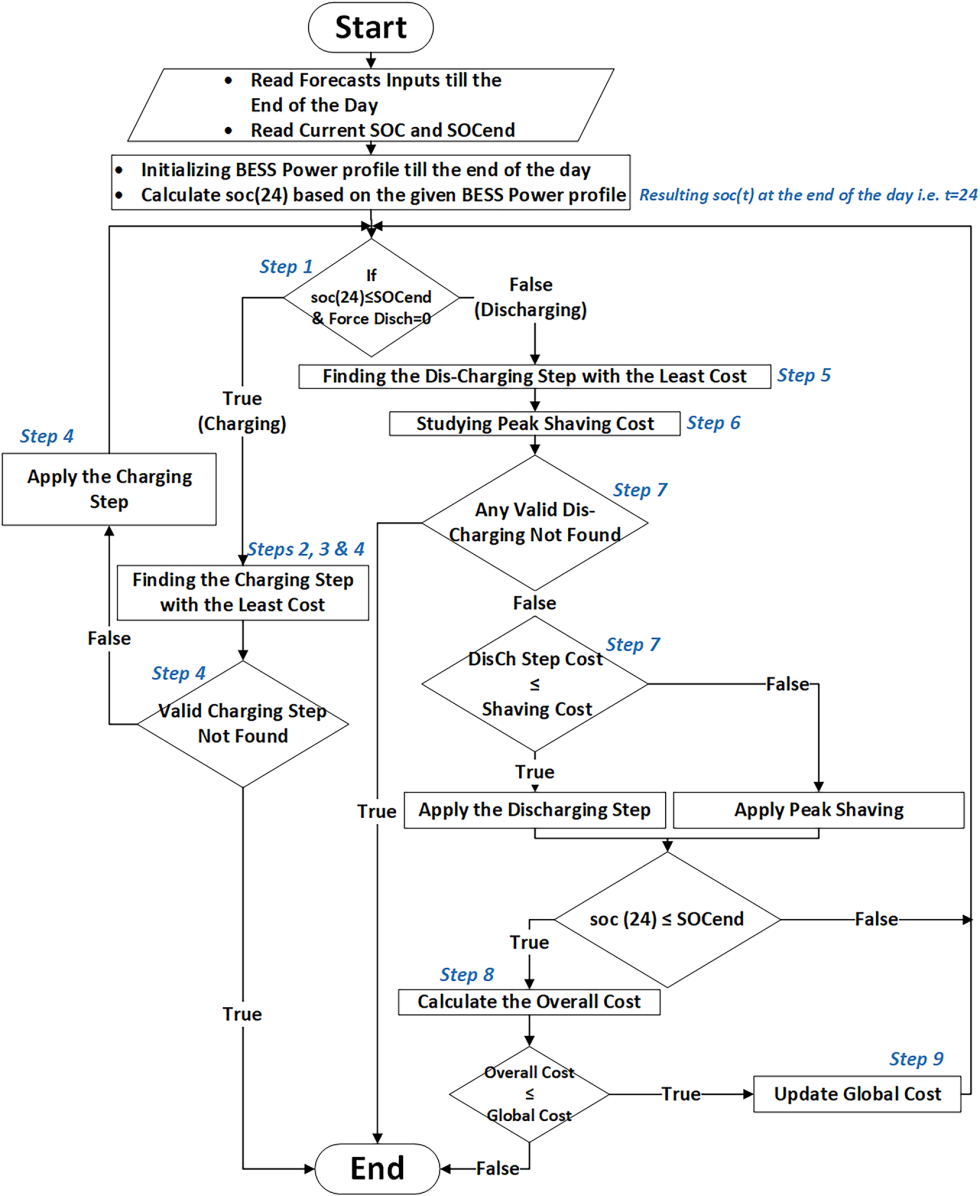
Flow chart of the proposed optimal BESS scheduling algorithm.

This part of the paper addresses how the overall optimization problem formulated in (2)-(15) will be solved using the proven DP technique, following the proposed solution algorithm expressed in Fig. 4.

The DP technique aims to simplify a complicated problem by breaking it down into simpler sub-problems and recursively solving them. DP is superior at breaking apart and recursively finding decisions that span several points, such as finding the optimal full-day-ahead battery power profile. The developed DP-based algorithm results and performance will be demonstrated to outperform the literature’s NAA, PSO and DE algorithms in [29]. The developed DP-based algorithm is different from the discussed DP-based algorithms in [22, 23, 27] in the formulation of discretizing the main problem to serve to optimize the comprehensive cost function (2) during a competitive execution time using data sampling of nine minutes.

The main idea beyond the proposed solution algorithm is as follows:

Given an initial condition of $({P}_{BESS}\left(t\right)=0 \forall {t}_{0}\le t\le 24)$, the idea is that the problem will be discretized into sub-problems of finding the best time slot (t) to insert a square pulse value of battery power (${P}_{step}$) having the least cost among all time slots in the last given $({P}_{BESS}\left(t\right))$ battery power profile, whether this ${P}_{step}$ has a positive/charging value or negative/discharging value.

The algorithm always tries to get the battery SOC at the end of the day at 12:00 AM with an adjustable setpoint (${SOC}_{end}$) to meet the following day’s load requirements. If the (${SOC}_{start}$) is lower the (${SOC}_{end}$) by an amount equivalent to several charging steps, the algorithm performs repetitive charging steps (Steps 2 to 4) till the end of the day batteries SOC $(SOC(24))$ reaches (${SOC}_{end}$). Then, the algorithm operates back and forth as a step–charging and then step-discharging until there is no cost minimization.

Algorithm execution steps can be summarized according to the flow chart in Fig. 4 as follows:

**Step 1:** With an initial (${P}_{BESS}(t))$, the algorithm calculates $(SOC(24))$. Depending on this SOC, the algorithm determines whether to insert a charging or discharging step $({P}_{step})$ to achieve the constraint in (5).

**Step 2:** In the step-charging mode, the algorithm first studies the cost of adding ($${P}_{step})$$ in each time slot (t) in terms of battery degradation. As the degradation cost depends on the DOD, each $${(P}_{step})$$ degradation cost is calculated at each time slot (t). Each time slot degradation cost calculation is done according to the whole day ahead new BESS power profile ($${P}_{BESS}\left(t\right)+{P}_{step}*f(t)$$) according to Eq. (8).

**Step 3:** Any time slot having its ($${P}_{step})$$ causes exceeding SOC limits as in (4), its cost will be infinite. The slot cost will also be infinite if it violates BESS power limits in (3). The charging algorithm also sets the infinity cost value to all time slots that the discharging algorithm previously chose. If the charging slot introduced a new peak value in $${(P}_{optimized}(t))$$ as in (14), and this peak value is greater than the ($${P}_{shaving\_limit})$$, the DCT additional cost is added to this slot cost.

**Step 4:** In this step, energy prices are added to all slots’ prices as (${P}_{step}*\Delta T*RTP(t)$). Finally, each time slot’s cost is evaluated such that the optimization may stop if all time slots are invalid (they all have infinite costs). If not, the charging algorithm chooses the time slot having the least cost to finally insert the (${P}_{step}*f(t)$) at.

**Step 5:** When $$SOC(24)$$ is re-evaluated after charging, and the algorithm determines to insert a discharging $${(P}_{step})$$, similar procedures to (Steps 2, 3 and 4) are executed to choose the least costly time slot to insert the ($${P}_{step})$$. This $$({P}_{step})$$ is likely to be inserted during high RTP periods. This will not be executed until it is compared with the next step.

**Step 6:** To serve peak shaving to reduce demand charges, several battery power steps all have a total energy value equal to the discharging step energy (${P}_{step}*\Delta T$) are inserted at the given $({P}_{optimized}\left(t\right))$ peak duration to flatten the curve to a certain shaving peak, this shaving peak must be greater than or equal to the (${P}_{shaving\_limit}$) shaving limit.

**Step 7:** At the last stage of step-discharging. (Steps 5 and 6) are evaluated such that the optimization may stop if all time slots are invalid (they all have infinite costs). If not, the discharging algorithm chooses the discharging behavior with the least cost, whether the step-discharging in the high tariff periods (Step 5) or the load shaving (Step 6). As a remark that will be demonstrated in (Example 2), the comparison between (Steps 5 and 6) contributed to determining the optimal peak, which is finally applied by the algorithm. This optimal peak can be greater than or equal to the (${P}_{shaving\_limit}$) shaving limit.

**Step 8:** To evaluate the combination of the last discharging step with the previous charging step, an overall cost calculation (cost of new ${P}_{optimized}(t)$) is performed after finishing discharging. The optimization stops when this cost exceeds the overall cost at the previous charging/discharging cycle.

**Step 9:** The algorithm restarts from (Step 1) after updating ${(P}_{BESS}(t))$ only if the current overall cost (calculated in Step 8) is less than the previous global cost so that there is room for further optimization.

## Description of the case study

**Fig. 5:**
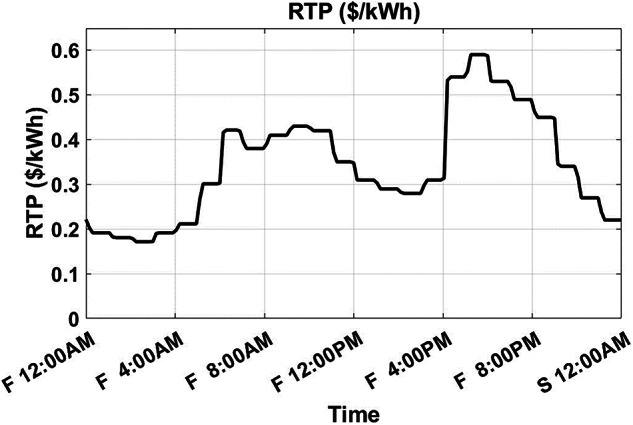
RTP profile along the day.

Several simulation examples are studied to show the proposed algorithm’s effectiveness. These examples are based on the MG in [29], where a 4 kW-capacity rooftop solar panel and BESS are utilized. The same parameters and scheduling circumstances in this study, such as the 28th-day index in the month and disregarding the battery degradation cost, are in effect. The RTP tariff is shown in Fig. 5. Specifications of the BESS are shown in Table 2. The DCT is set to be $8.03/kW/month.

**Table 2:** BESS model.

Parameter	Value
Rated Power	4 kW
Energy Capacity	8kWh
Initial and Final SOC (SOC_end_)	0.15 at 12AM
Charging/Discharging Efficiency	0.9
SOC_min_	0.1
SOC_max_	0.9

Since the DCT is based on calculating the moving average power over the last ten minutes [29, 30], the data sampling period should be less than ten minutes. Also, 5-min sampling will create an over-sampling problem, leading to slower execution. A nine-minute sampling interval (*ΔT* = 0.15 h) is chosen for the optimization problem, resulting in a total of 160 time slots per day ($t$ = 0: *ΔT*:24). On the other hand, PV output power predicted profiles should also follow this sampling period to detect rapid shading profile effects.

Therefore, the algorithm optimizes 160 discrete decision variables, each representing the battery power (${P}_{BESS}\left(t\right))$ at a specific time slot. Each decision variable can take on discrete values in steps of (${P}_{step}=100W$), within the range of − 4 kW (discharging) to + 4 kW (charging), i.e., up to 40 levels per time slot.

The simulations run on Matlab 2021a with an Intel(R) Core (TM) i7-10750H CPU @ 2.60 GHz processor and 16 GB memory. FIT will not be considered, and battery temperature is assumed to be ideal.

This paper considers four examples. The first example demonstrates the feasibility of the proposed algorithm. The second example shows the compromise between RTP and DCT. The third example considers all costs (RTP, DCT, and degradation). In contrast, the fourth example discusses the effect of selecting various algorithm parameters on the final optimized cost observed in the third example.

Different terms used in the following examples are defined as follows:

- Profits percentage (%) is defined as normalized cost savings before and after using BESS.
- Average BESS degradation cost ($/kWh) is the degradation cost ($${C}_{BESS}$$) divided by the total discharged BESS energy units over a certain period.
- Average BESS cost ($/kWh) is the unit cost of BESS utilization and is calculated as the sum of both the degradation cost ($${C}_{BESS}$$) and the BESS charging cost due to RTP ($) divided by the total discharged BESS energy units over a certain period.
- Average BESS profit ($/kWh) is the unit profit of BESS utilization and will be calculated as the sum of both the BESS discharging profit due to RTP ($$\text{\$}$$) and the profit due to DCT reduction divided by the total discharged BESS energy units over a certain period.
- The payback period is the number of months that will return the capital cost of BESS ($${C}_{Initial}$$) through the optimized BESS monthly profits.

## Results and discussion

To validate the superiority of the developed algorithm, it is compared against the NAA algorithm results from [29], using the same parameters, constraints, and the same unoptimized net power profile (${P}_{feeder}\left(t\right)$). In Examples 1 and 2, battery degradation is not considered (as in [29]), while in Examples 3 and 4, it is included. The day index refers to the position of the day within the billing cycle of a calendar month. For example, day index = 1 corresponds to the first day of the month, while day index = 28 refers to the 28th day. This distinction is important because the DCT is typically calculated based on the highest recorded peak demand within the billing month. Therefore, the daily RTP and degradation costs should be weighted by the number of remaining days in the month and combined with the DCT cost to yield the overall comprehensive monthly price. In Example 1, the optimization is performed on day index = 28 to demonstrate the algorithm’s ability to minimize cost while taking into account previously recorded peak loads. In all other examples, the optimization is carried out assuming day index = 1, where no prior peak demand has occurred. The summarized results are presented in Table 3 and illustrated in the following examples.

**Table 3:** Australian case study results and comparison summary.

	Without BESS	BESS-NAA in [29]	Developed BESS algorithm			
Example number	–	–	1	2	3	4
Day index	–	28^th^	28^th^	1^st^	1^st^	1^st^
Degradation considered	–	No	No	No	Yes	Yes
Initial and final SOC	–	0.15	0.15	0.15	0.15	0.20
Cost due to DCT ($)/month	17.16	13.65	13.65	15.20	16.46	15.25
Cost due to RTP ($)/day	3.25	1.91	− 0.1	− 0.31	− 0.36	− 0.34
Profit (%)	0	23.7	33.6	94.8	89.0	89.6
Average BESS degradation cost ($/kWh)	–	–	–	–	0.016	0.016
BESS degradation cost ($) /Day	–	–	–	–	0.23	0.23
Average BESS cost ($/kWh)	–	–	0.250	0.240	0.260	0.260
Average BESS profit ($/kWh)	–	–	0.750	0.500	0.500	0.500

### A. Example 1: proposed algorithm benchmarking

The recorded historical maximum power consumption on the 28th day of the current month is 1.7 kW, which is the peak shaving limit $({P}_{shaving\_limit})$. The NAA algorithm demonstrated effective load peak shaving, ensuring the peak did not exceed 1.7 kW. However, this resulted in higher RTP costs than those presented in this section.

**Fig. 6:**
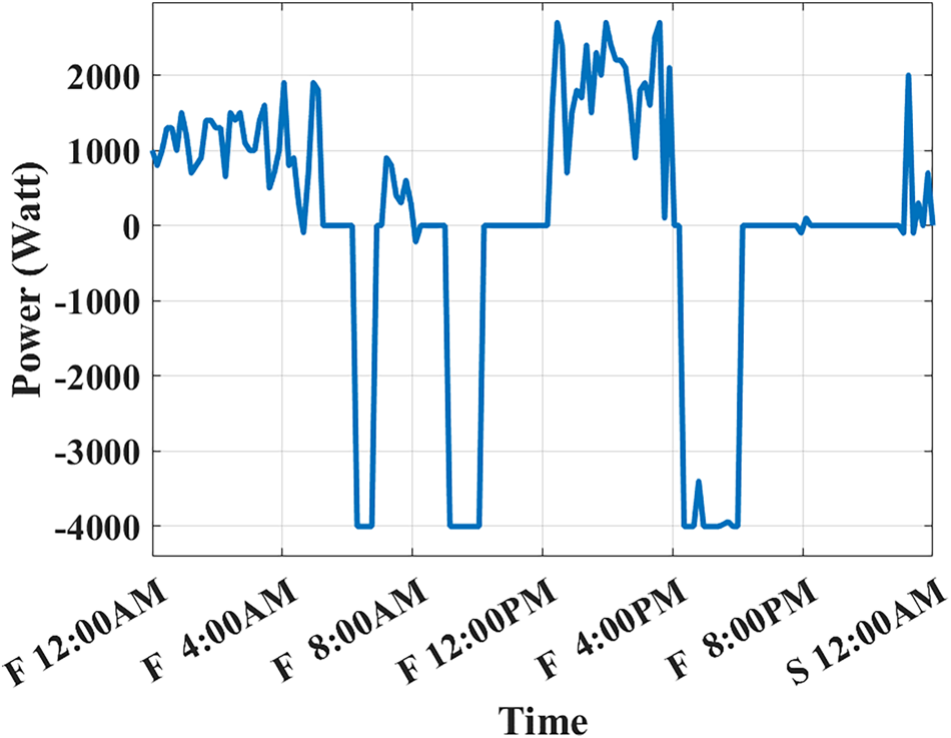
Schedule using the proposed algorithm considering a given historical peak.

**Fig. 7:**
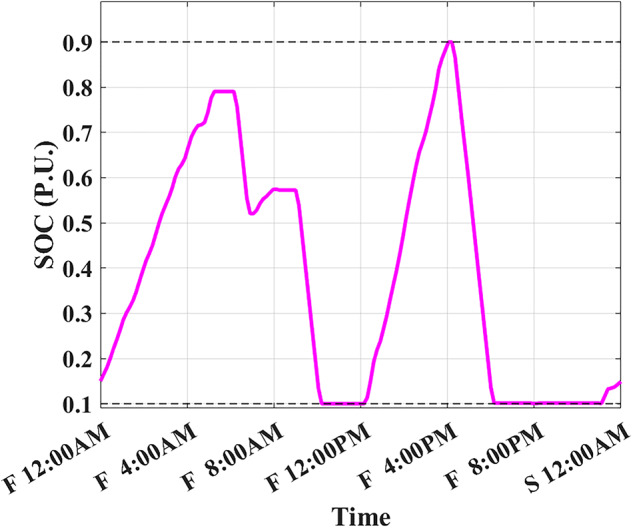
SOC using the proposed algorithm considering a given historical peak.

The developed algorithm follows the same parameters and constraints as the literature case study. Figures 6,7 show the developed BESS schedule and SOC. The algorithm utilizes all low RTP cost hours to charge the BESS up to not exceeding the shaving limit value $({P}_{shaving\_limit})$ in the optimized net power profile (${P}_{optimized}(t)$), then discharges the BESS at maximum power during high RTP cost hours. Figure 7 shows two complete charge/discharge cycles within the day.

**Fig. 8:**
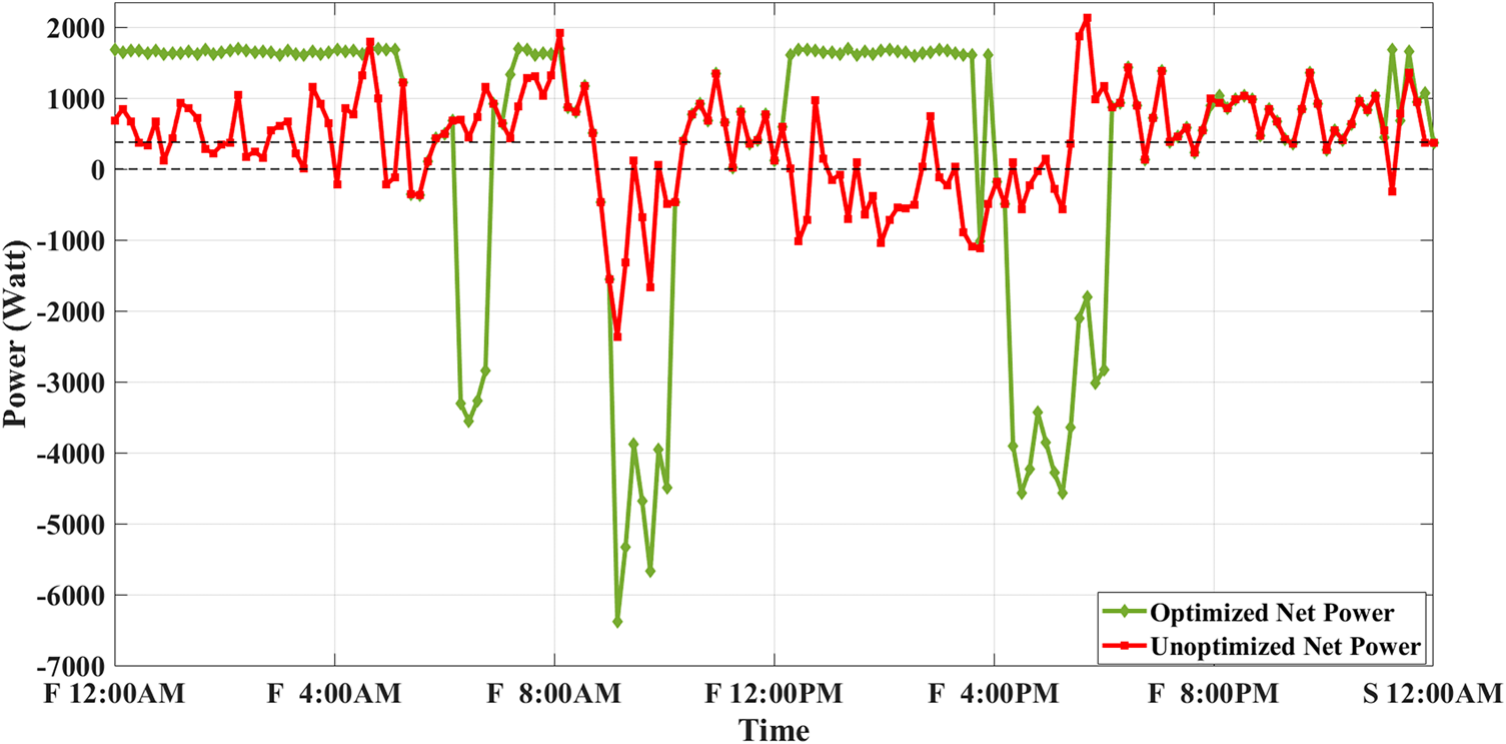
Optimized net power using the developed algorithm considering a given historical peak.

Figure 8 shows the overall unoptimized net power profile before using BESS (${P}_{feeder}\left(t\right)$) and the overall optimized net power profile after using BESS (${P}_{optimized}(t)$).

With only three days remaining in the current month, the algorithm has retained the previously recorded peak of 1.7 kW as the final optimized peak. This decision is based on the analysis that any RTP cost savings from surpassing this peak would be multiplied by only three, resulting in less benefit than the additional DCT penalty incurred. Consequently, the developed algorithm limits the daily peak value to 1.7 kW, optimizing RTP costs while maintaining the same DCT costs as reported in the literature. The average cost of using BESS is primarily driven by RTP charging costs, while profits are derived from RTP discharging and peak shaving, reducing the peak from 2.1 kW to 1.7 kW.

The results demonstrate that the developed algorithm not only reduces the peak value effectively but also achieves a lower average RTP cost compared to the NAA algorithm. This highlights the potential for significant cost savings and improved efficiency in BESS management.

### D. Example 2: compromising between costs due to RTP and DCT

As demonstrated in Step 7 of the algorithm, this simulation aims to highlight a key contribution of the developed algorithm: its ability to balance the optimization of two cost terms in (2), namely RTP costs and DCT costs. The algorithm determines the extent of peak shaving and BESS discharging during high RTP cost periods.

**Fig. 9:**
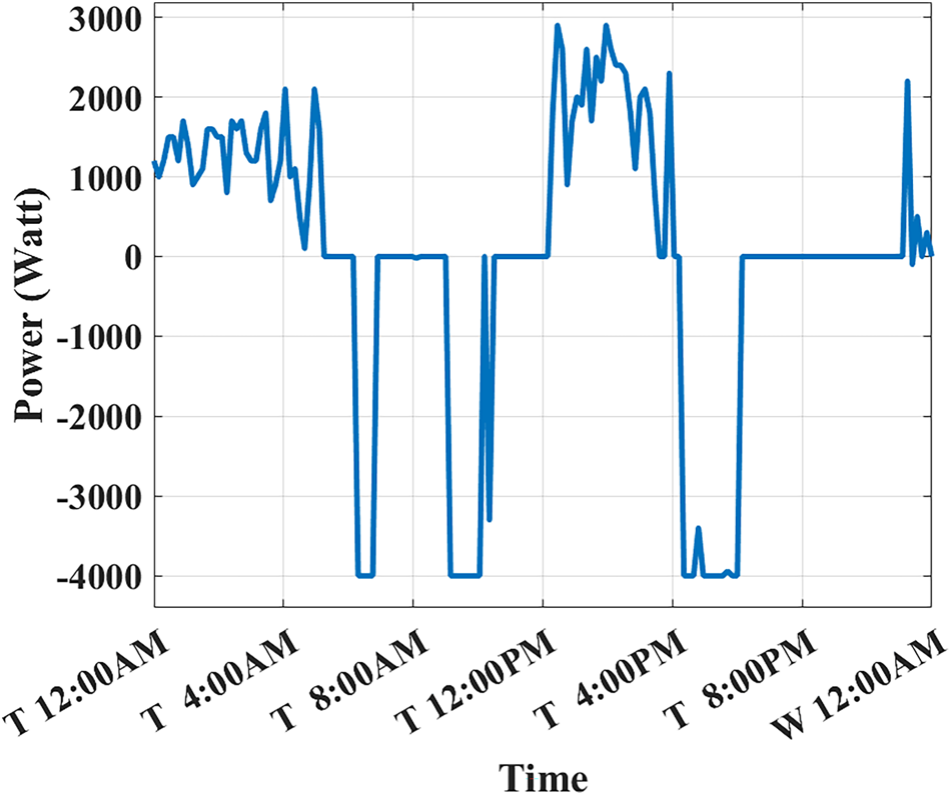
Schedule with optimal peak.

**Fig. 10:**
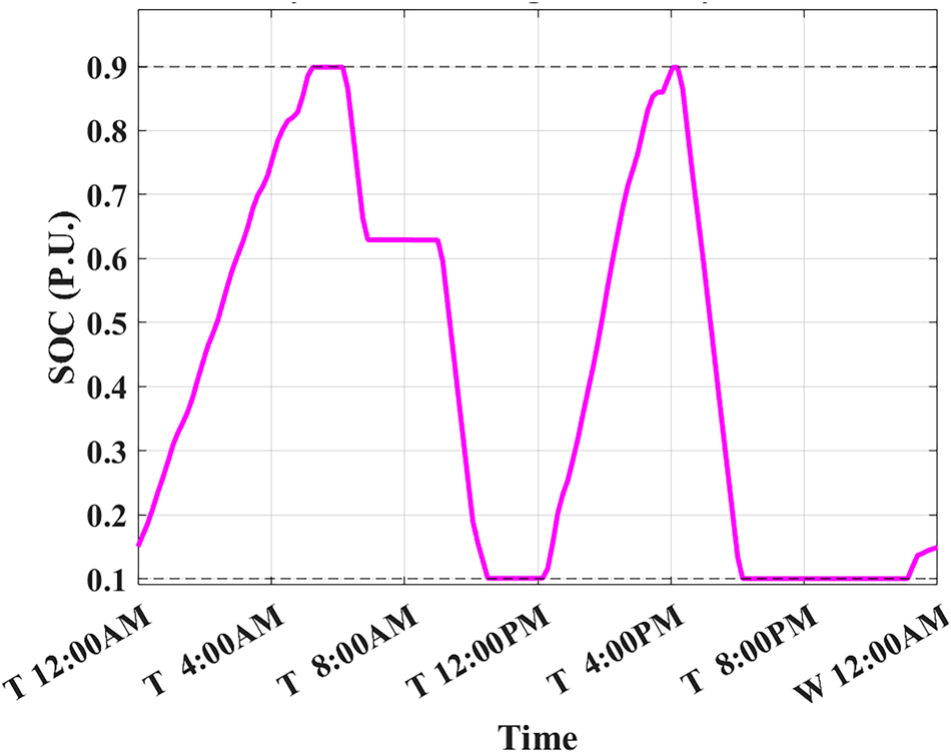
SOC with optimal peak.

**Fig. 11:**
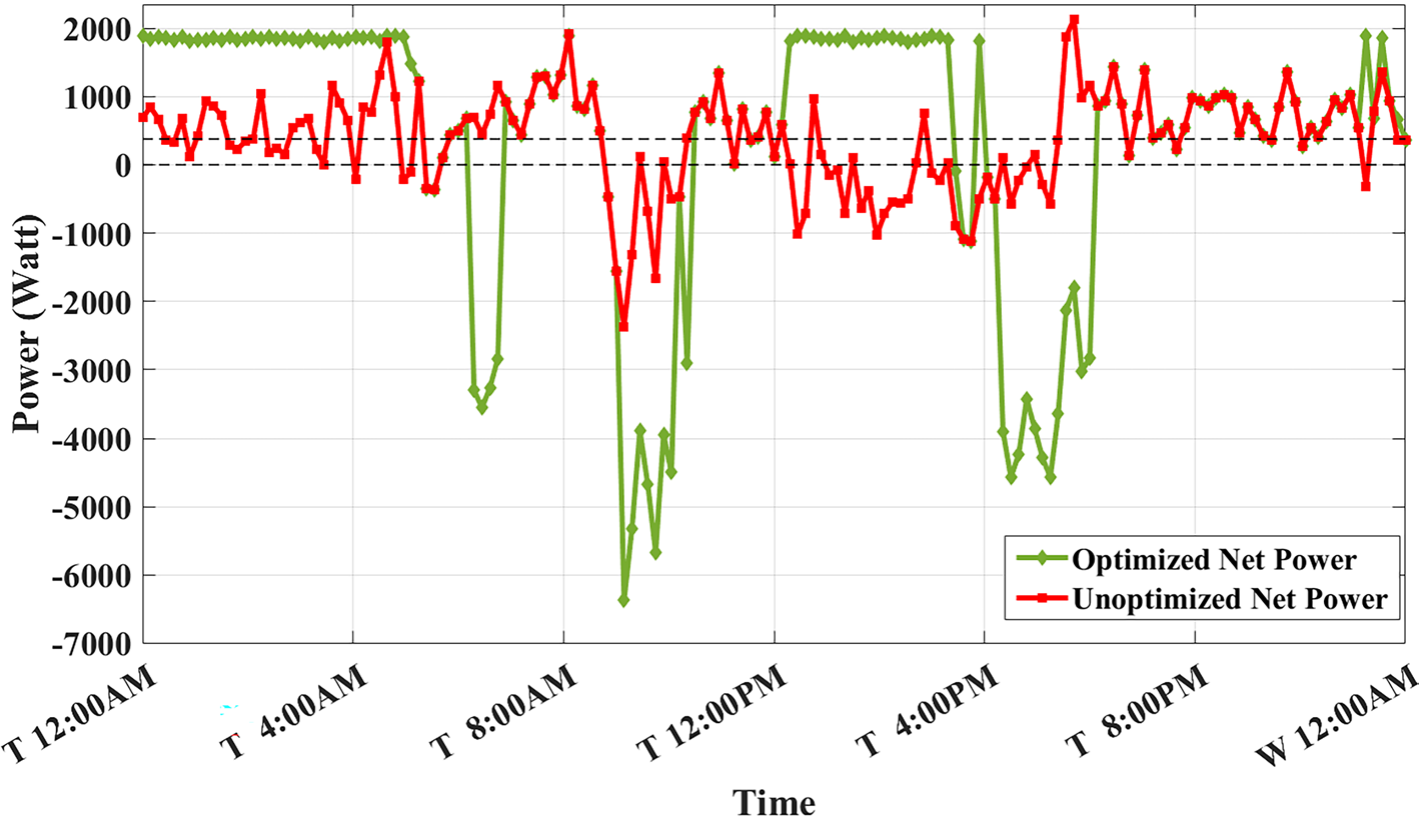
Optimized net power with optimal peak.

Assuming a repetitive daily load profile for an entire month, the total monthly cost is calculated as the daily RTP cost multiplied by 30, plus the DCT cost. Figures 9,10 illustrate the developed BESS schedule and SOC. Figure 11 shows the algorithm’s contribution in selecting an optimal peak of 1.895 kW, which is higher than the peak in Example 1. This higher peak broadens the potential savings in RTP costs over the month.

The results of this scheduling indicate that the average cost of using BESS is driven solely by RTP charging costs, while the average profit from BESS usage comes primarily from RTP discharging profits.

### E. Example 3: considering all cost terms of RTP, DCT and battery degradation

One of the main contributions of this study is the simultaneous consideration of all three cost terms: RTP, DCT, and battery degradation. The inclusion of battery degradation as a factor in the optimization process is crucial for accurately assessing the true costs and benefits of BESS usage.

**Fig. 12:**
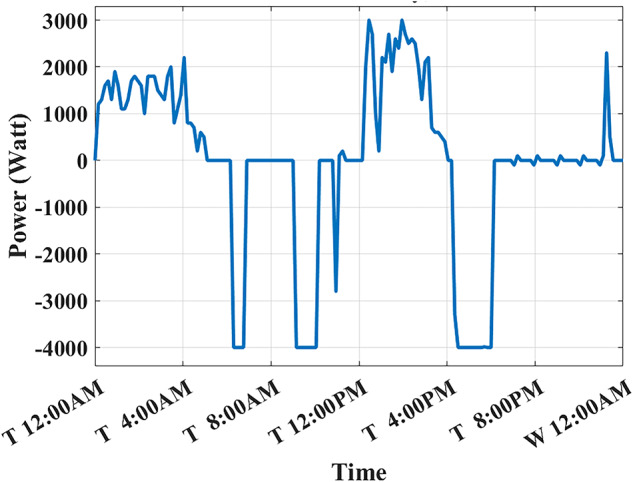
Schedule considering battery degradation.

**Fig. 13:**
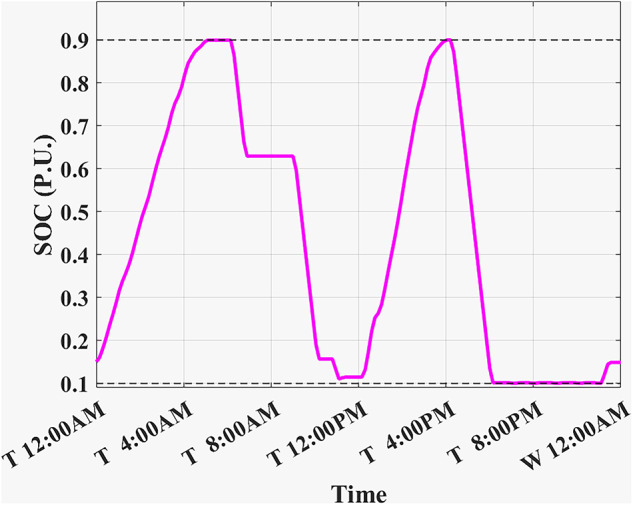
SOC considering battery degradation.

**Fig. 14:**
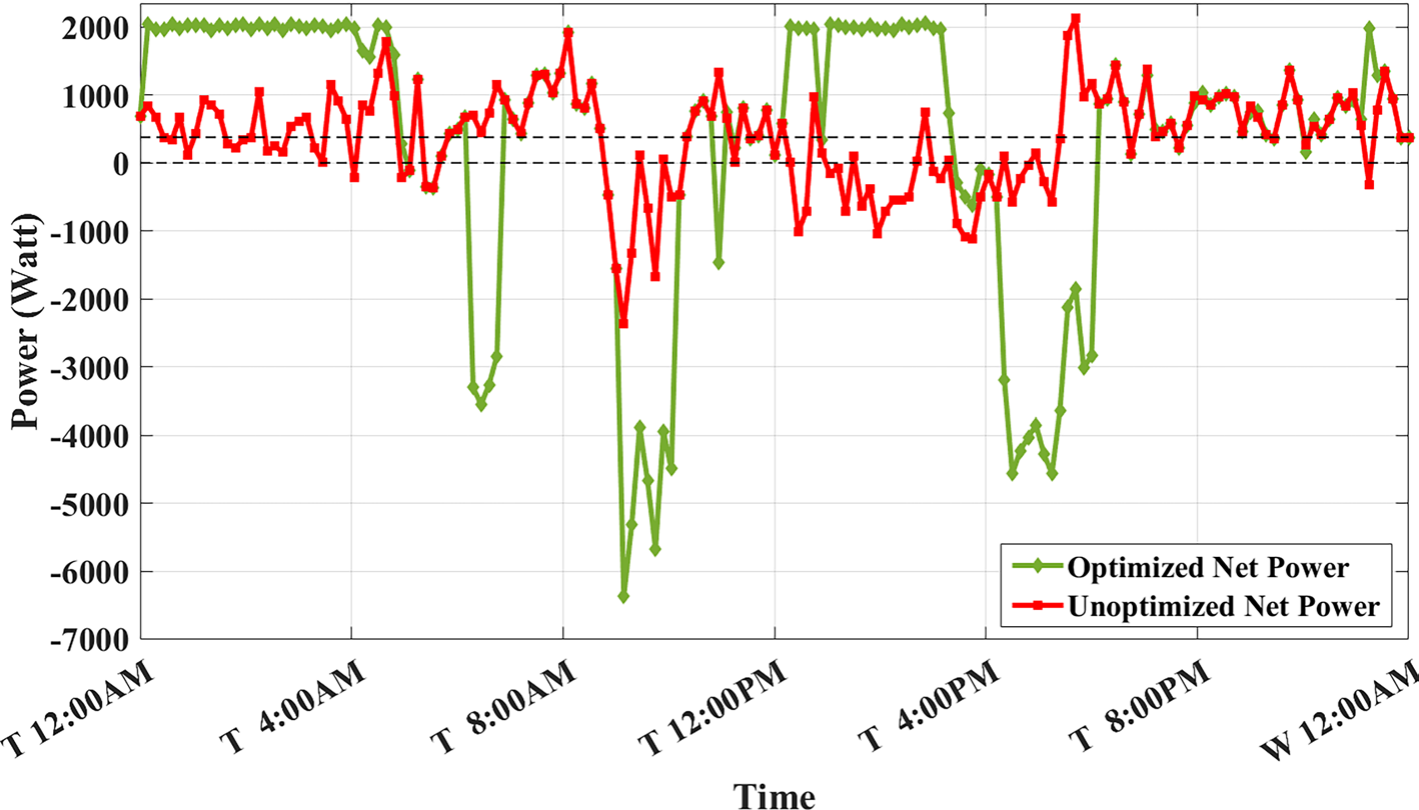
Optimized net power considering battery degradation.

In this example, the total monthly cost to be minimized is calculated as the sum of the daily RTP cost multiplied by 30, the daily BESS degradation cost multiplied by 30, and the DCT cost. Figures 12,13 illustrate the developed BESS schedule and SOC. Figure 14 shows how the algorithm selected the optimal peak of 2.05 kW, allowing for greater RTP cost minimization throughout the month compared to the two previous examples.

The results of this scheduling indicate that the average cost of using BESS arises from RTP charging costs and degradation costs, while the average profit comes from RTP discharging profits.

### F. Example 4: sensitivity analysis on different parameters

#### (1) Solution step (${P}_{step}$) sensitivity on execution time:

**Fig. 15:**
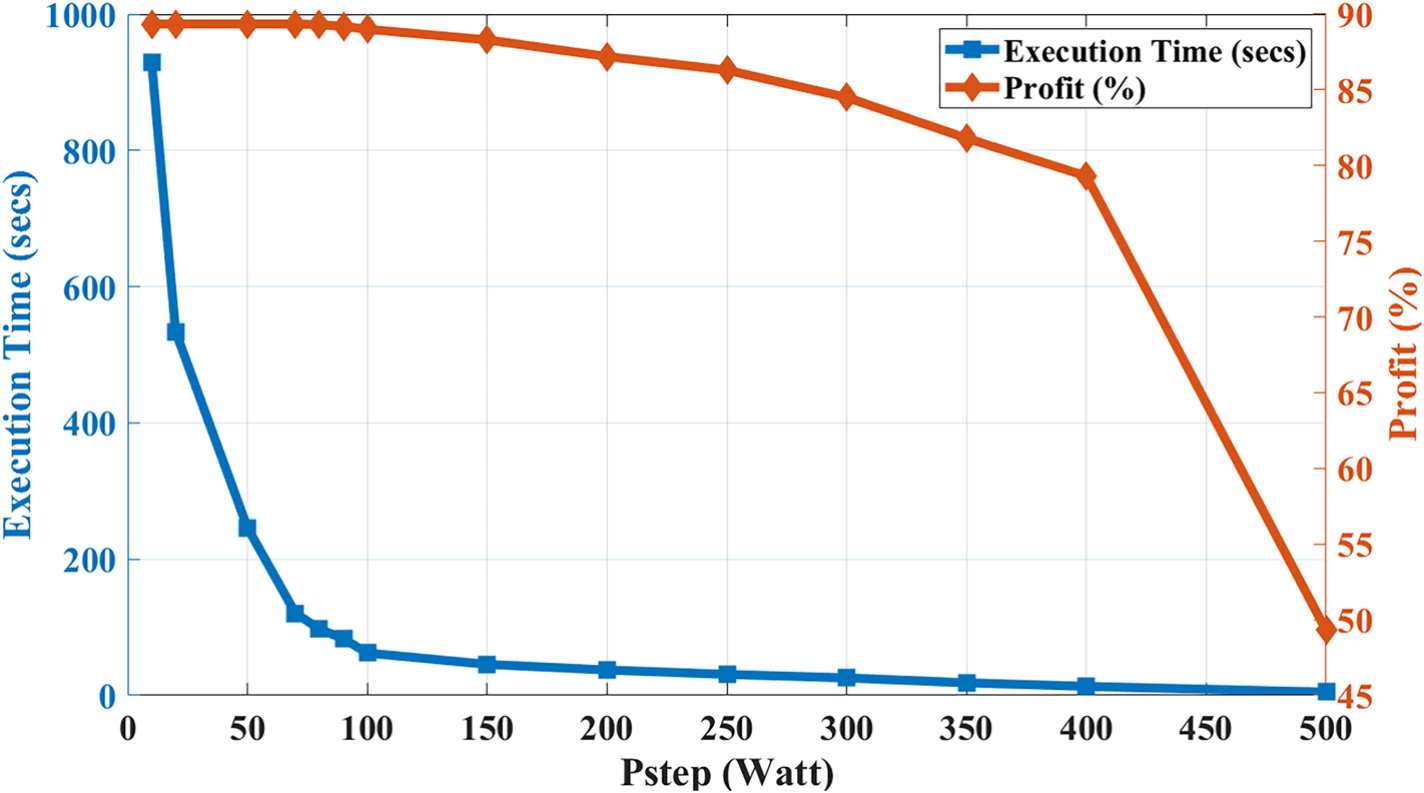
Execution time (secs) and profit (%) versus different Pstep values.

In this analysis, a study of algorithm execution time (in seconds) and profits (in percentage) versus different ${P}_{step}$ values are implemented in (Example 3), considering the sampling period remains nine minutes. Figure 15 illustrates the variation effect of the solution step (${P}_{step}$). The less this solution step is, the more profits the algorithm will get as it will have a better resolution but at the cost of more execution time and vice versa. While decreasing ${P}_{step}$ at low values, profit will get saturated without optimization but with much more execution time.

Therefore, selecting the ${P}_{step}$ parameter value is crucial. This paper chose this parameter to be 100 watts, resulting in an execution time of 62 s and profits of 89%, as demonstrated in Table 3. Hence, this competent execution time is significantly less than the sampling period (*ΔT*), contributing to a responsive online accommodation to input predicted profiles error corrections without losing any future scheduling horizon points.

For example, if new predicted input data—such as updated prices, loads, or PV—becomes available during the day, the BESS scheduling module recalculates the optimal battery power profile for the remaining time slots. Thanks to the algorithm’s fast execution, the updated schedule is applied in the very next time slot, ensuring no delay or loss of optimization following the input data correction.

#### (2) BESS initial and final SOC setpoints sensitivity:

**Fig. 16:**
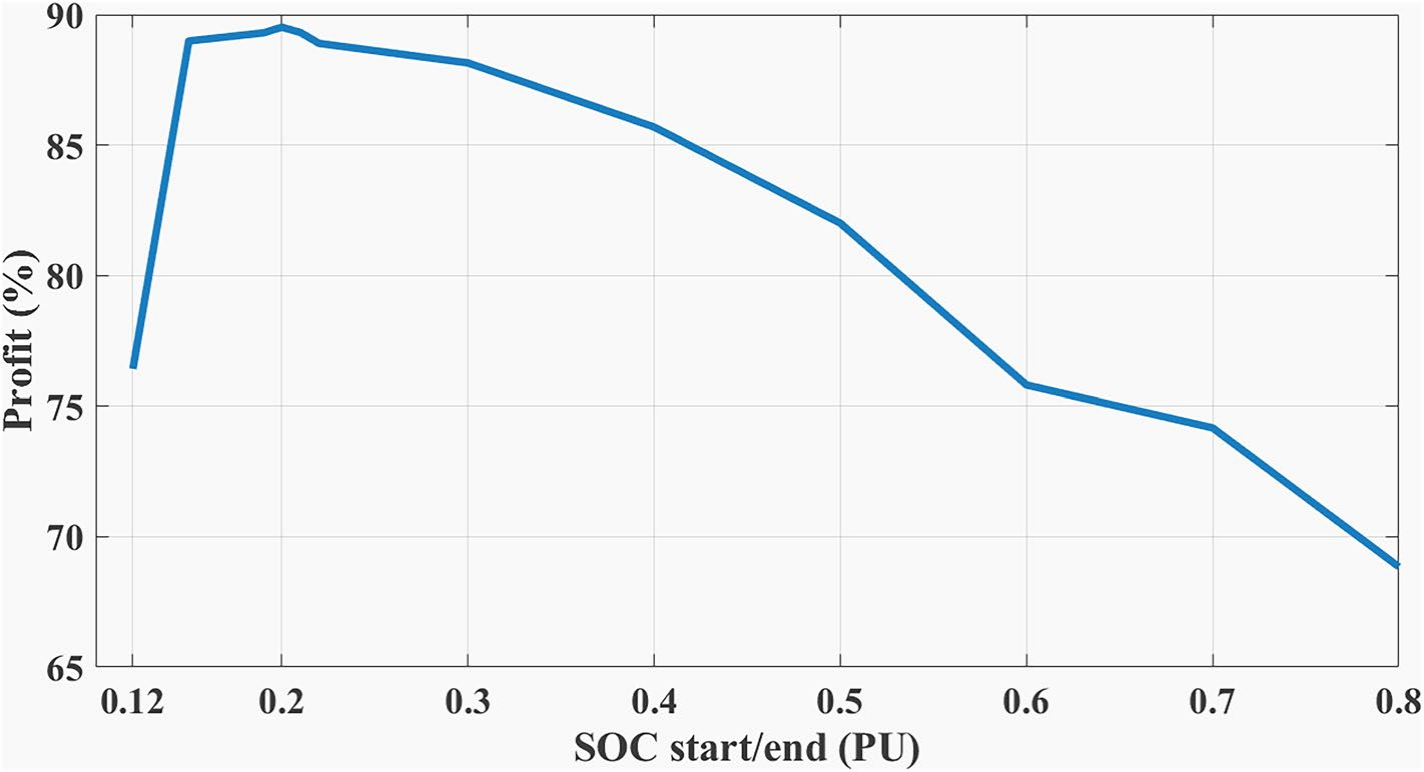
Different SOC setpoints versus profits for the studied load profile.

**Fig. 17:**
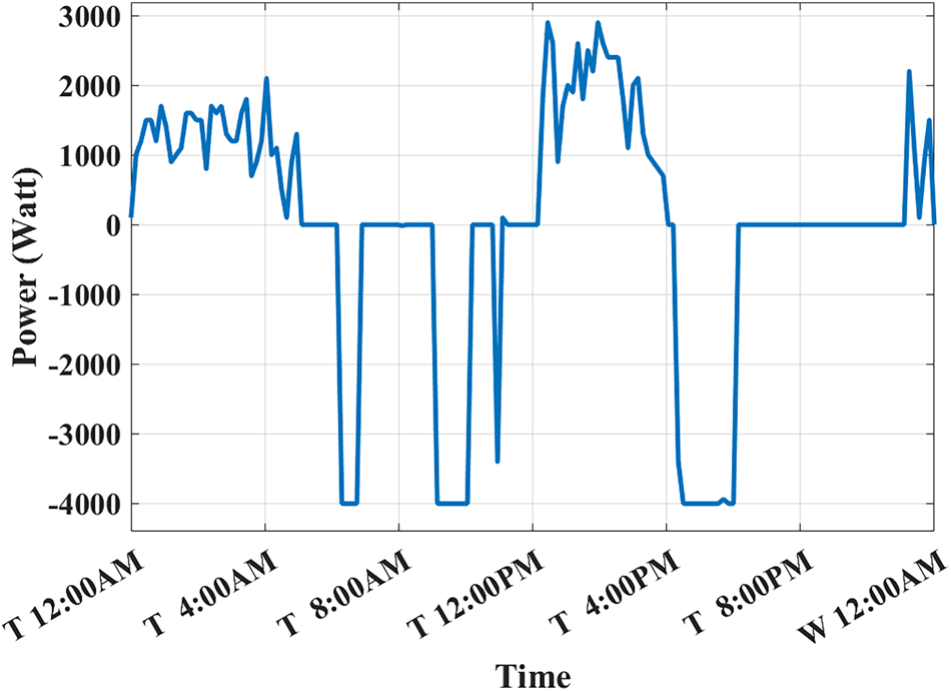
Schedule with SOC_start/end_ = 0.2

**Fig. 18:**
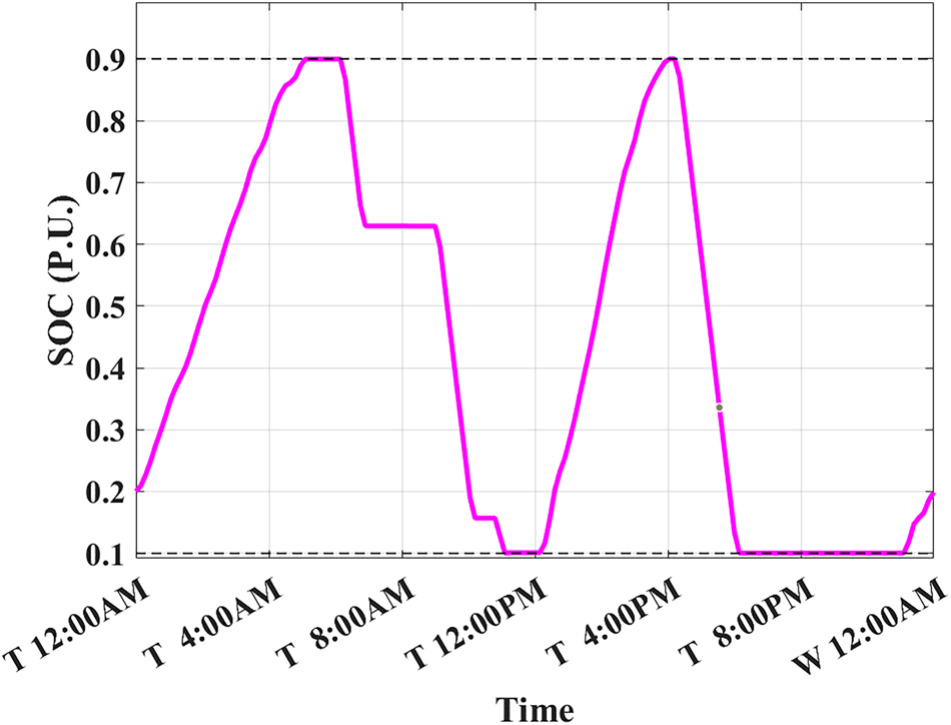
SOC with SOC_start/end_ = 0.2

**Fig. 19:**
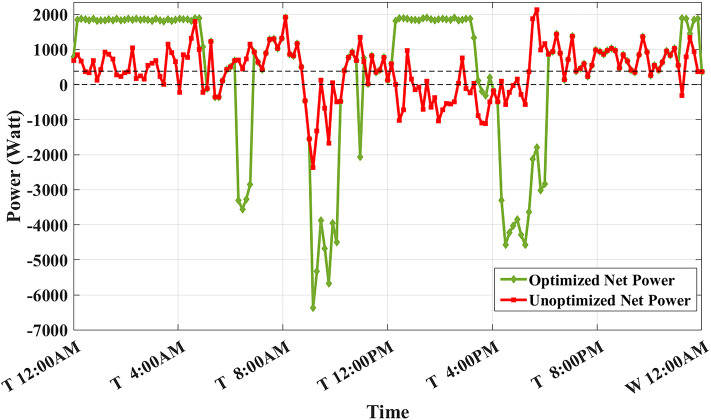
Optimized net power with SOC_start/end_ = 0.2

The choice of BESS SOC setpoint at the end of the day (${SOC}_{end}$) is critical as it affects the final profit according to load profile, BESS parameters, and degradation model. When studying (Example 3) but with different (${SOC}_{end}, {SOC}_{start}$) such that (${SOC}_{end}={SOC}_{start}$). Figure 16 shows the best SOC that the system should have at 12:00 AM is 0.20. Using this setpoint, BESS scheduling results are shown in Figs. 17, 18. Then, the BESS charged during the low RTP price period at the beginning of the day till 5:00 AM with less charging power achieving less (${P}_{optimized}(t)$) peak of 1.9 kW, as shown in Fig. 19.

#### (3) BESS capacity sensitivity:

**Fig. 20:**
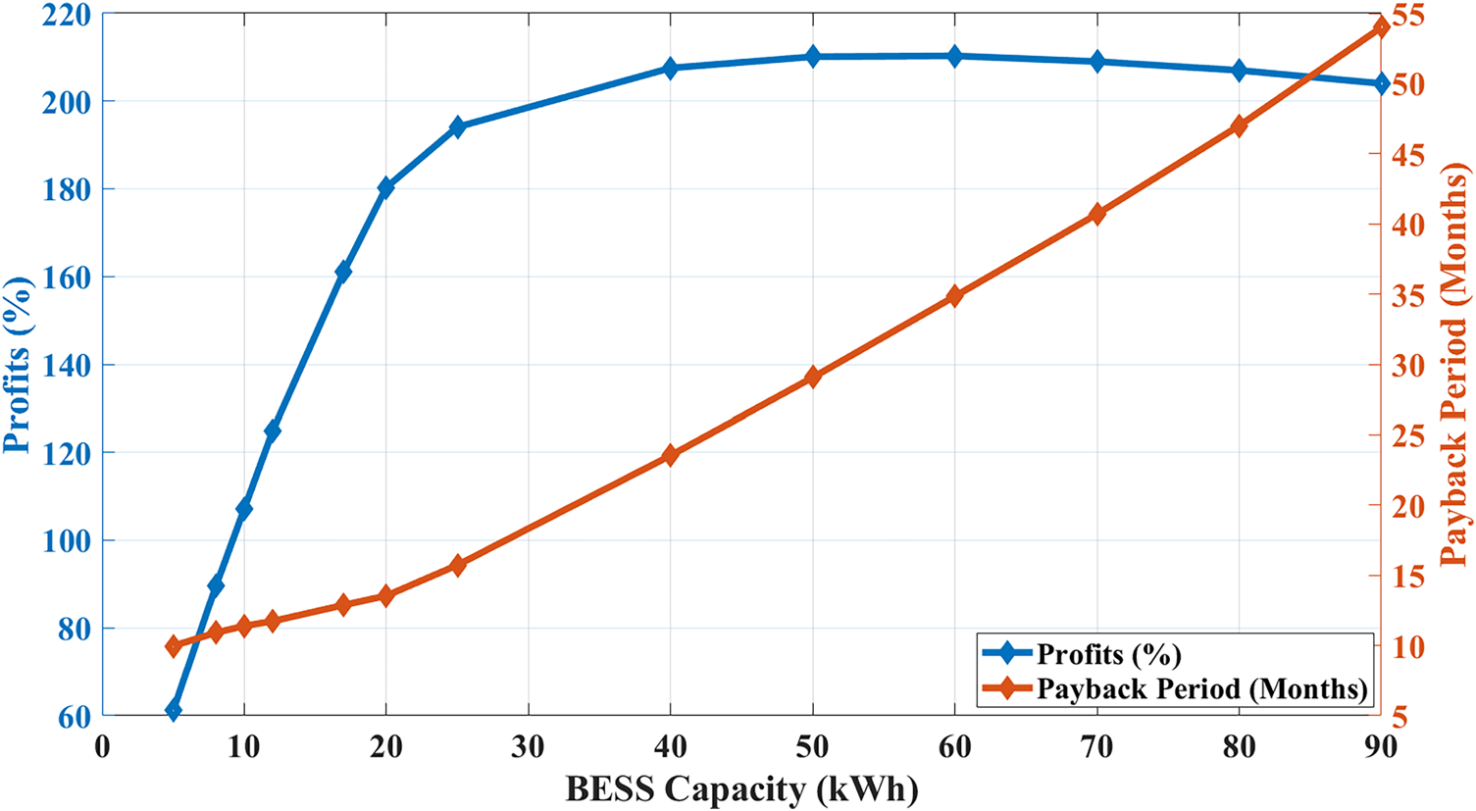
BESS installed capacity versus profits and payback period.

When studying (Example 3) with different BESS capacities, Fig. 20 describes the effect of changing the installed nominal capacity on the final profit and payback period. With low capacities of up to 20kWh, the more capacity is installed, the more profits the algorithm will collect due to RTP, which will increase the cost due to DCT. Afterward, the more capacity is added up to 50kWh, the less battery degradation cost will become because the optimum ${SOC}_{start/end}$ setpoint will be around 0.5, allowing the operation of the BESS to be around its optimum SOC level [35]. The optimized peak reaches 4.85 kW when using a capacity of 50kWh. Next, the more capacity is added, the same scheduling results will be generated with the same 4.85 kW peak and 0.5 ${SOC}_{start/end}$ setpoint but with a higher BESS unit cost, which finally reduces the overall profit.

Table 3 summarizes all the different examples discussed in the Australian case study, considering all the parameters and constraints in the literature, as indicated in Table 2.

## Conclusions and future research

This paper introduced a novel and comprehensive cost function that incorporates three different cost terms for optimization. The developed optimal BESS scheduling algorithm, based on DP, was evaluated through a case study in Australia. Using a nine-minute data sampling interval to detect and shave load peaks, the algorithm achieves a significantly reduced execution time—averaging around one minute—enabling efficient intra-day operation and adaptability to prediction errors. In Australia, the BESS unit cost is $0.260/kWh, which is relatively inexpensive compared to the utility’s RTP range of $0.190 to $0.590/kWh. Running the algorithm over a month with a repetitive daily load profile effectively minimizes costs related to RTP and DCT while accounting for the BESS degradation. This optimization of the overall cost function yields a monthly profit of approximately 90%, amounting to $103, relative to an initial BESS cost of $1120.

Multiple algorithm simulations are conducted against different installed BESS nominal capacities and different SOCs at the beginning and end of the day ($$SO{C}_{start} and SO{C}_{end})$$. Hence, these offline simulation results show optimal values of installed capacity, $$SO{C}_{start} and SO{C}_{end}$$ for the studied MG load profiles and pricing parameters. Moreover, as previously specified in (Section “Methodology and approach”), the proposed algorithm is computationally intensive and not suitable for single-board computers, which could pose limitations in terms of cost and platform simplicity. Other limitations are left for future research, such as:

- Proposing the prerequisite STLF module algorithm to be integrated with the proposed battery scheduling module and the SCADA platform in [31, 32], to validate the complete hardware implementation using the integration model shown in Fig. 2.
- Optimization in a FIT metering topology.
- Incorporating more realistic operational degradation factors, including actual battery operating temperature, real-world depth of discharge, and effective lifecycle behavior, rather than relying solely on calculated estimates used in this study.

## Supplementary Information

Supplementary Information.

## Data Availability

All data generated or analyzed during this study are included in this published article as supplementary MATLAB figures containing all datasets, which are enumerated and presented exactly as described in the article.

## Constants

*a, b, c*: Coefficients for (13) [-]
$${C}_{Initial}$$: Batteries capital cost [$]
*DCT*: Monthly demand charge tariff [$/kW/month]
$${E}_{BESS nominal}$$: Batteries nominal capacity [kWh]
$${{P}_{BESS}}_{max}$$: Maximum battery charging power [W]
$${{P}_{BESS}}_{min}$$: Minimum batteries discharging power [W]
*RTP(t)*: Real-time pricing energy tariff [$/kWh]
$$SO{C}_{max}$$: Maximum allowed batteries state of charge [-]
$$SO{C}_{min}$$: Minimum allowed batteries state of charge [-]
$$SO{C}_{start}$$: Initial battery state of charge at the instance of algorithm execution [-]
$${\mu }_{ch-disch}$$: Batteries charging/discharging efficiency [%]
$$f\left(t\right)$$: A pulse function of unit magnitude at the time (t) for a duration of *ΔT* [-

## Parameters

${P}_{step}$: A step value of battery power [W]
$SO{C}_{end}$: Final battery state of charge at the end of the day at 12:00AM [-]
*ΔT*: Sampling period of all profiles [0.15 h]
*Δt*: Discrete-time interval between consecutive measurements [-]
${t}_{0}$: Scheduling execution starting time [-]

## Variables

$${C}_{BESS}$$: BESS degradation cost [$]
$${C}_{Demand}$$: Cost due to *DCT* of monthly peak demand [$]
$${C}_{Energy}$$: Cost due to *RTP* or energy billing function [$]
*Discharge First Mode*: A flag that reverses algorithm charging/discharging cycling [-]
*Discharge Flag(t)*: A flag time series that prevents step-charging at the same time slots of previously decided step-discharging [-]
*DOD(t)*: Batteries depth of discharge [-]
$${E}_{BESS}(t)$$: Batteries stored energy [kWh]
$${E}_{BESS D\_capacity}(t)$$: Degraded battery capacity [kWh]
*Force Disch*: A flag that forces the algorithm to the step-discharging path [-]
*N(t)*: Batteries remaining life cycles number [-]
$${P}_{average}$$: The average of the net power profile before applying BESS [W]
$${P}_{BESS}(t)$$: Optimized BESS power profile respective to the AC side [W]
$${P}_{feeder}(t)$$: Net power before applying BESS [W]
$${P}_{feeder\_peak\_f}$$: Current month forecasted load peak [W]
$${P}_{HPV}(t)$$: Forecasted photovoltaic power profile respective to the AC side [W]
$${P}_{load}(t)$$: Netload profile, including feeders’ losses [W]
$${P}_{optimized}(t)$$: Net power profile after applying BESS [W]
$${P}_{optimized\_his}$$: Past recorded peak in the current month [W]
$${P}_{shaving\_limit}$$: Shaving threshold limit specified by the forecasting module [W]
$$SOC(t)$$: Batteries state of charge [-]
$$t$$: Time slot [hour]
